# Rose Bengal-Mediated Photoinactivation of Multidrug Resistant *Pseudomonas aeruginosa* Is Enhanced in the Presence of Antimicrobial Peptides

**DOI:** 10.3389/fmicb.2018.01949

**Published:** 2018-08-20

**Authors:** Joanna Nakonieczna, Katarzyna Wolnikowska, Patrycja Ogonowska, Damian Neubauer, Agnieszka Bernat, Wojciech Kamysz

**Affiliations:** ^1^Laboratory of Molecular Diagnostics, Department of Biotechnology, Intercollegiate Faculty of Biotechnology, University of Gdańsk and Medical University of Gdańsk, Gdańsk, Poland; ^2^Department of Inorganic Chemistry, Faculty of Pharmacy, Medical University of Gdańsk, Gdańsk, Poland; ^3^Laboratory of Experimental Embryology, Institute of Genetics and Animal Breeding, Polish Academy of Sciences, Warsaw, Poland

**Keywords:** antibiotic resistance, *Pseudomonas aeruginosa*, photoinactivation, CAMEL, pexiganan, XDR, MDR, antimicrobial peptides

## Abstract

Due to the overuse of antibiotics in medicine and food production, and their targeted mechanism of action, an increasing rate in spreading of antibiotic resistance genes has been noticed. This results in inefficient therapy outcomes and higher mortality all over the world. *Pseudomonas aeruginosa* (carbapenem-resistant) is considered one of the top three critical species according to the World Health Organization’s priority pathogens list. This means that new drugs and/or treatments are needed to tackle infections caused by this bacterium. In this context search for new/alternative approaches that would overcome resistance to classical antimicrobials is of prime importance. The use of antimicrobial photodynamic inactivation (aPDI) and antimicrobial peptides (AMPs) is an efficient strategy to treat localized infections caused by multidrug-resistant *P. aeruginosa*. In this study, we have treated *P. aeruginosa* cells photodynamically in the presence and in the absence of AMP (CAMEL or pexiganan). The conditions for aPDI were as follows: rose bengal (RB) as a photosensitizing agent at 1–10 μM concentration, and subsequent irradiation with 514 nm-LED at 23 mW/cm^2^ irradiance. The analysis of cell number after the treatment has shown that the combined action of RB-mediated aPDI and cationic AMPs reduced the number of viable cells below the limit of detection (<1log_10_ CFU/ml). This was in contrast to no reduction or partial reduction after aPDI or AMP applied separately. Students *t*-test was applied to test the statistical significance of the results. Noteworthy, our treatment proved to be effective against all 35 clinical isolates of *P. aeruginosa* tested within this study, including those characterized as multiresistant. Moreover, we demonstrated that such treatment is safe and does not violate the growth dynamics of human keratinocytes (77.3–97.64% survival depending on the concentration of the studied compounds or their mixtures).

## Introduction

Since the discovery of penicillin in 1929, followed by a great success in the control of bacterial infections, a golden era of antibiotics has begun. This successful era lasted for many years, during which whole-cell screens, search for broad-spectrum natural and synthetic antibacterial drugs, were provided to the market and to the clinics. Later on, due to the increase in bacterial resistance to conventional drugs, the search turned into more targeted molecules, which, however, did not bring much success in recent years. Nowadays, we are facing the problem of global resistance and failure in antimicrobial drug discovery (Brown and Wright, 2016). Of particular concern are infections caused by the ESKAPE pathogens, which include ***E****nterococcus faecium*, ***S****taphylococcus aureus*, ***K****lebsiella pneumoniae*, ***A****cinetobacter baumannii*, ***P****seudomonas aeruginosa*, and ***E****nterobacter* spp. (Rice, 2008).

*Pseudomonas aeruginosa* is a chief opportunistic pathogen that can cause nosocomial infections in susceptible persons in medical institutions. This bacterium can spread *via* human-to-human direct distribution, and also *via* water systems (up to 50%) in hospital wards (Blanc et al., 2004). In the hospitals, it was isolated from various medical devices, sanitary installations, but also from flowerpots (D’Agata, 2014). *P. aeruginosa* is responsible for the complicated infections, particularly in people with compromised immunity, e.g., oncological patients, people after transplantation, elderly people, that are frequently hospitalized. This bacterium causes skin and soft tissue infections, which can be fatal for people with burns and after surgeries. Mortality among *P. aeruginosa*-infected patients is estimated at 20%, but it can reach as high as 50%, e.g., in the case of placenta infection (Lautenbach et al., 2010; Ceniceros et al., 2016). The most dangerous population among *P. aeruginosa* isolates constitute those producing metallo-β-lactamases, conferring resistance to all penicillins, cephalosporins, and carbapenems. The latter has been known as the last resort drugs in the treatment of Gram-negative infections (Potron et al., 2015). Much attention has nowadays been paid to the development of strategies that can lower the use of antibiotics and slow down the spread of the resistance phenomenon. Being in-line with this trend, photodynamic inactivation of multiresistant pathogens has emerged as a promising alternative to antibiotics.

Antimicrobial photodynamic inactivation (aPDI), also known as photoantimicrobial chemotherapy (PACT), relies on the action of three elements: a small-molecular-weight chemical compound (photosensitizer, PS), light, and oxygen. Light irradiation activates PS, which leads to the generation of singlet oxygen (energy transfer) and/or oxygen radicals (electron transfer). All the reactive oxygen species (ROS) generated during aPDI are responsible for cytotoxic effect toward bacterial cells due to inactivation of proteins, lipids, and nucleic acids. Because of multitargeted action of ROS, acquiring resistance to this form of antibacterial treatment is highly unlikely and has not been experimentally confirmed so far (Giuliani et al., 2010; Tavares et al., 2010; Wainwright et al., 2017). Another advantage of PDI includes double selectivity based on the local delivery of a PS and light, that both need to act concomitantly to produce ROS. Such a local delivery of a PS and light allows avoiding systemic exposure and potential adverse effects of the treatment. Practically, every living microorganism can be inactivated by means of aPDI. Often the presence of bacterial cells is not sufficient to trigger disease, and the damage to host cells is caused by various virulence factors produced by the pathogen. aPDI has been shown to efficiently reduce virulence factors which seems to be a rational approach to control infection (Fila et al., 2017). It was found, however, that the efficacy of photoinactivation of Gram-negative species is less efficient as compared to Gram-positive ones, due to the presence of an outer membrane, which constitutes a natural barrier limiting a simple diffusion of a PS (Bertoloni et al., 1990). This means, that *in vivo*, a high concentration of a PS and irradiance have to be delivered to obtain satisfactory clinical outcome (Hashimoto et al., 2012; Fila et al., 2016). Thus in the case of *in vivo* treatment, the danger of cyto- and/or phototoxicity exists toward host eukaryotic tissues, resulting from higher light doses and/or higher PS concentration applied to photoinactivate Gram-negative bacteria. These might include damage to biomolecules leading to the breakdown of cell structure, and damage to organelles, as well as initiation of necrotic or apoptotic pathways. Various approaches have been reported to literature to overcome the problem of lower PDI efficacy toward Gram-negative bacteria as compared to Gram-positive ones, e.g., addition of positive charge to a PS (Hamblin et al., 2002; Tegos et al., 2006). Also, polymyxin B addition to anionic or neutral porphyrins enabled to carry out photoinactivation of Gram-negative species (Nitzan et al., 1992).

Another approach to diminish the negative effects of high doses of PS and light is to decrease the effective concentration of PS by combining its action with antimicrobial peptides (AMPs). AMPs have been studied in the context of combating drug-resistant pathogens as an encouraging alternative to classical antimicrobials. They are part of the innate immune system and act against diverse-spectrum microorganisms. AMPs are generally amphipathic and have a length of up to 50 amino acids. Their net positive charge and a high proportion of hydrophobic residues mediate binding to anionic groups on the bacterial cell surface and facilitate interaction with membranes to produce lethal pores (Zasloff, 2002). The bacterial surface has a net negative charge due to the presence of lipoteichoic acids and lipopolysaccharides as opposed to sphingomyelin, phospholipids, and cholesterol in the membrane of eukaryotic cells. Such differences between bacterial and eukaryotic membrane composition are the basis of selectivity for AMPs activity.

There have been several studies on the subject of combining the action of aPDI with AMP through producing conjugates of a photosensitizer (mostly porphyrin derivatives, or xanthenes) with various peptides (Bourre et al., 2010; Yang et al., 2011; Liu et al., 2012; Dosselli et al., 2013, 2014; Johnson et al., 2013; Costley et al., 2017; Le Guern et al., 2017; Tsuchikama et al., 2017). However, the subject of a simple combination of two kinds of the molecules was underinvestigated. Such an approach is simpler and also cheaper as the conjugation step can be avoided. There has been only one elegant work done recently on the combination of three types of a photosensitizer, namely curcumin, methylene blue, and chlorin Ce6 with an AMP aurein 1.2, where free, non-conjugated molecules showed excellent activity (de Freitas et al., 2018). In the cited work, the authors focused on Gram-positive species, namely *Enterococcus faecium*, with an unknown antibiotic resistance pattern. In this work, we have been investigating Gram-negative species *P. aeruginosa*, which is currently considered a species of critical priority. As a photosensitizer for our analysis we have chosen rose bengal (RB) – 4,5,6,7-tetrachloro 2′,4′,5′,7′-tetraiodo derivative of fluorescein, that is known to mainly generate singlet oxygen upon visible light exposure. RB possess a strong absorption band at 550 nm, and strong molar absorption coefficient (95,000 dm^3^ mol^−1^ cm^−1^) (Ludvikova et al., 2016). RB properties have been extensively investigated in technology known as photochemical tissue bonding in tissue repair (Gu et al., 2011).

In our research, we have investigated the new potential therapy that could be applied in the treatment of local infections caused by multidrug-resistant *P. aeruginosa*. We examined the combined action of rose-bengal-based aPDI and two AMPs, namely pexiganan and CAMEL, showing significantly enhanced effect of the studied combination. Moreover, we investigated our approach toward the wide repertoire of clinical isolates of *P. aeruginosa*, including carbapenem-resistant strains, proving that the proposed approach is a viable antimicrobial strategy. Our extensive analysis revealed that such a new approach was not toxic to human skin keratinocytes, nor did it affect their growth dynamics.

## Materials and Methods

### Bacterial Strains and Growth Conditions

The reference *P. aeruginosa* ATCC^®^ 10145 strain was used in the study. The 35 clinical *P. aeruginosa* isolates, were provided by Julianna Kurlenda and isolated from patients hospitalized in the Provincial Hospital in Koszalin and the Provincial Hospital in Gdansk, and Sebastian Mucha from Independent Public Clinical Hospital No. 1 of Silesian Medical University in Katowice (**Table 1**). Bacterial cultures were grown aerobically in LB medium (Carl Roth, Karlsruhe, Germany) at 37°C with shaking (150 rpm) or on LB agar (Sigma-Aldrich, Munich, Germany). Antibiotic susceptibility for piperacillin (PIP); ticarcillin-clavulanic acid (TIM); piperacillin-tazobactam (TZP); cefepime (FEP); ceftazidime (CAZ); imipenem (IPM); meropenem (MEM); doripenem (DOR); gentamicin (GEN); amikacin (AMK); netilmicin (NET); tobramycin (TOB); ciprofloxacin (CIP); levofloxacin (LVX); colistin (CST); polymyxin B (PMB); fosfomycin (FOF); aztreonam (AZT) was determined by the Vitek 2 system, AST-N331 cards (bioMérieux, Craponne, France). The Vitek 2 minimum inhibitory concentration (MIC) results were interpreted using an Advanced Expert System according to Clinical and Laboratory Standards Institute (CLSI) recommendations (CLSI, 2012). Categorization of resistant, intermediate resistant, susceptible *P. aeruginosa* was determined according to the guidelines of the European Centre for Disease Prevention and Control (ECDC) and the Center for Disease Control and Prevention (CDC). The description of strains as multidrug-resistant (MDR) was defined as acquired non-susceptibility to at least one agent in three or more antimicrobial categories, extensively drug-resistant (XDR) was defined as non-susceptibility to at least one agent in all but two or fewer antimicrobial categories (i.e., bacterial isolates remain susceptible to only one or two categories) (Magiorakos et al., 2012).

**Table 1:** Drug resistance characteristics of *Pseudomonas aeruginosa* clinical isolates used in the study.

Strain		Source of isolation	PIP	TIM	TZP	FEP	CAZ	IPM	MEM	DOR	GEN	AMK	NET	TOB	CIP	LVX	CST	PMB	FOF	AZT	Resistance status
1	10145	ND																	R		
2	3042/s	Urine													R	R					
3	3318/p	Ear																	R		
4	2969/s	Wound																			
5	1561/o	Bronchi		R				R	R	R			R						R		MDR
6	3404/p	Urine		R															R		
7	3117/s	Abdomen																	R		
8	3146/s	Wound																	R		
9	4190/p A	Wound	R	R	R										IR	R			R		MDR
10	1959/o	Bronchi	R	R	R		R	R	R	R					IR	R			R		XDR
11	3109/o	Urine	R	R	R	R					R	R		R	R	R					MDR
12	1286/o	Urine, blood						R	R	R									R		
13	55/K	Bronchi	R	R	R	R		R	R	R	R	R	R	R	R	R			R		XDR
14	978/K	Pleura	R	R	R	R	R	R	R	R					R	R			R		XDR
15	K3	Blood	R	R	R		R	R	R	R									R		MDR
16	K4	Blood	R	R	R	R	R	R	R	R	R	R	R	R	R	R			R		XDR
17	792/K	Wound						R	R	R	R	R	R						R	IR	MDR
18	393/K	Urine						R	R	R									R	IR	
19	133/K	Urine																			
20	23/K	Urine		R															IR		
21	887/K	Wound	R	R	R	R	R	R	R	R						R			R	R	XDR
22	169/K	Nose	R	R	R	R		R	R	R	R	R	R	R	R	R				IR	XDR
23	3752/sz	Ear		R																	
24	4908/p	Urine		R															R		
25	3907/sz	Uterus						R	R	R									R		
26	15/p I	Tracheostomy tube																	R		
27	149/p	Urine																			
28	153/s	Urine		R															R		
29	547/p	Ear																	R		
30	1885/K	Bronchi	R	R	R	R		R	R	R	R	R	R	R	R	R			IR		XDR
31	556/K	Wound		R			R	R	R	R	R	R	R	R	R	R			IR		XDR
32	1651/p	Urine																	R		
33	1304/s	Urine	R	R	ND		R												R		MDR
34	1804/p	Urine																	R	IR	
35	2284/p	Ear		R															IR		
36	143/p	Wound		R															R		

*Antimicrobial susceptibility testing included following antimicrobials: PIP, piperacillin; TIM, ticarcillin-clavulanic acid; TZP, piperacillin-tazobactam; FEP, cefepime; CAZ, ceftazidime; IPM, imipenem; MEM, meropenem; DOR, doripenem; GEN, gentamicin; AMK, amikacin; NET, netilmicin; TOB, tobramycin; CIP, ciprofloxacin; LVX, levofloxacin; CST, colistin; PMB, polymyxin B; FOF, fosfomycin; AZT, aztreonam. R, resistant; IR, intermediate resistant, no letter – sensitive.*

### Chemicals

The photosensitizer used in the study was rose bengal (RB, 4,5,6,7-tetrachloro-2′,4′,5′,7′-tetraiodofluorescein, SigmaAldrich, Munich, Germany). The prepared stock solution of 10 mM RB was diluted with a sterile double distilled water and kept at 4°C for up to a month. All the reagents used for the synthesis were of pure analytical grade purchased from Sigma-Aldrich, Germany.

### Peptides Synthesis

Peptides: CAMEL (CAM; KWKLFKKIGAVLKVL-NH_2_) and pexiganan (PEX, GIGKFLKKAKKFGKAFVKILKK-NH_2_) were synthesized by solid-phase method using Fmoc (9-fluorenylmethyloxycarbonyl) chemistry on a Polystyrene Amide AM-RAM resin as described previously (Sikora et al., 2018). The peptides were purified by Reverse-Phase High-Performance Liquid Chromatography (RP-HPLC), and then freeze-dried. The mobile phase was water and acetonitrile containing 0.1% TFA (v/v). UV detection at 214 nm was used. The identity of the compounds was confirmed using mass spectrometry (ESI-MS). The compounds were analyzed by HPLC in the following conditions: 20–80% acetonitrile (0.1% TFA), 10 min.

Molecular weights of peptides were as follows: CAM – 1770.3 g/mol; PEX −2477.2 g/mol.

### Determination of CAM, PEX MIC/MBC Values

Minimum inhibitory concentration (MIC, the lowest concentration of AMP that inhibited bacterial growth) toward *P. aeruginosa* 10145 was assessed as follows: a total of a 100 μL of bacterial suspension in brain-heart infusion medium (BHI, bioMérieux, Carpenne, France) was placed in 96-well plate. The inoculum of bacteria was 10^4^ CFU in each well. A 100 μL of twice diluted AMP CAM or PEX was added to wells (concentration range 0–512 μg/mL). The cells were incubated 24 h at 37°C. The MBC (minimum bactericidal concentration) values were assessed as a minimal concentration of AMP that caused 99.9% death of bacterial cells (Cockerill et al., 2012).

### Photoinactivation Experiments

Bacterial strains were cultured in 5 mL of LB medium for 18–22 h at 37°C with agitation (250 rpm). Cells were diluted with a fresh broth to the density of 0.2 McFarland units (10^7^ CFU/mL). A total of 100 μL of each culture was loaded into a 96-well plate and incubated in the dark at 37°C for 15 min, either with or without the addition of RB. The concentrations of RB used in the study were 1–100 μM. Accordingly, in the photoinactivation experiments with the use of AMPs, PEX, or CAM was added along with RB and incubated in the dark at 37°C for 15 min. To remove unbound compounds, cells were washed three times with 1 mL of sterile PBS. Samples were irradiated with a total fluence of 15, 30, and 60 J/cm^2^ (duration of irradiation 668, 1335, and 2668 s, respectively). For the irradiation procedure, LED lamps (SecureMedia, Poland), designed and produced for the Laboratory of Molecular Diagnostics, emitting green light were used (λ_max_ 514 nm, FWHM = 33 nm: the width of a spectrum curve measured between those points on the *y*-axis which are half the maximum amplitude) (Ogonowska et al., 2018). The irradiance was 23 mW/cm^2^. The distance from a LED to an illuminated sample was 10 cm, and the power density measurement represents the value at the distance of 10 cm. Aliquots incubated in the dark with and without RB served as dark controls. Additionally, during each experiment light-only treatment was performed without the addition of RB. After irradiation, 10 μL aliquots were taken to perform 10-fold serial dilutions in PBS, ranging from 10^−1^ to 10^−4^. Ten microliter aliquots of each dilution were plated on LA plates (Carl Roth, Karlsruhe, Germany). Cells were incubated overnight at room temperature, and then, for 2–4 h at 37°C, the colonies formed were counted, and the results were analyzed statistically. Each experiment was performed in triplicate.

### Photosensitizer Uptake

Overnight cultures prepared as those for the photoinactivation experiments were diluted to 0.2 McFarland units. A total of 100 μL of culture was placed in Eppendorf tube and washed three times with PBS. After the last centrifugation step, cells were diluted in 100 μL of PBS. Then, RB was added to a final concentration of 1, 2, 5, and 10 μM and incubated for 15 min in the dark at 37°C. Next, cell suspensions were centrifuged (5 min, 3300 rcf) and supernatants were placed in a 96-well plate. Absorption was measured at λ = 565 nm. The cells treated similarly but without incubation with RB served as background control. The concentrations of RB accumulated were read based on calibration curves obtained from the measurements of free RB.

### Fluorescence Microscopy

Overnight cultures of *P. aeruginosa* were diluted 10-times (c.a. 3.1 McFarland units). A total of 100 μl of culture was placed in Eppendorf tube and washed with PBS 3 times, following 5 min centrifugation at 3300 rcf. Next, CAM, PEX and/or RB were added to a final concentration of 50 μM or 100 μM. Cells were incubated in the dark at 37°C for 5–30 min. The excess of the compounds was centrifuged (5 min, 3300 rcf) and the pellet was resuspended in 50 μL PBS. Four μL of bacterial suspension was placed on microscope glass. The observation was carried out under a fluorescence microscope (Olympus BX51, Hamburg, Germany) with the F-View Soft Imaging System digital camera. Excitation/emission spectra were λ_ex/em_ = 510–550 nm/>570 nm. At least three observations were made on three independent days.

### Photohemolysis Assay

A 2%-solution of sheep erythrocytes was prepared in PBS by diluting a 1 mL stock solution (100%) in 50 ml PBS. Erythrocytes were centrifuged (5 min, 3300 rcf), and resuspended in 50 mL of the RPMI-1640 medium (Sigma-Aldrich, Hamburg, Germany). A 400 μL of a 2% solution of erythrocytes were placed in Eppendorf tube, together with CAM, PEX and/or RB to a final concentration of 5 μM each. Cells were incubated in the dark for 5 or 15 min at 37°C. Then, cells were washed twice with PBS and suspended in a final volume of 400 μL. A 200 μL was transferred into 96-well plate and kept in the dark, the second half of the solution was irradiated (fluence: 30 J/cm^2^, irradiance: 23 mW/cm^2^). The plates were centrifuged (6 min, 3100 rcf), and supernatants were transferred to a new 96-well plate. The absorbance of released hemoglobin was measured at λ = 470 nm. A 10% SDS-treated erythrocytes were used as a control of complete hemolysis (100% hemolysis). The data were presented according to the equation:

% integrity=100 %−% hemolysis

Three independent experiments were performed and presented values are the mean ± SD.

### Photo- and Cytotoxicity Assay Based on MTT

HaCaT cells (CLS 300493) were seeded at the density of 1 × 10^4^ cells/well the day before treatment in three biological replicates for each condition in two 96-well plates (for light and dark conditions, respectively). Cells were grown in a standard humidified incubator at 37°C and 5% CO_2_ atmosphere in DMEM high glucose medium supplemented with 1mM sodium pyruvate, 1 mM non-essential amino-acids, 100 U/mL penicillin, 100 μg/mL -streptomycin, 2 mM glutamine, 10% fetal calf serum (all reagents from Life Technologies/Thermo Scientific). The compounds were added to the final concentration of 5 μM or 10 μM RB, 5 μM or 10 μM of AMP (CAM or PEX), or mixtures thereof. The compounds were added directly to the medium, and further incubated for 15 min at 37°C. Afterward, the cells were washed twice with PBS and finally dissolved in 100 μL PBS. Next, cells were subjected to illumination with 514 nm-light (irradiance: 23 mW/cm^2^, 22 min). Cell survival was measured after 24 h of incubation at 37°C by MTT [3-(4,5-dimethylthiazol-2-yl)-2,5-diphenyltetrazolium bromide] assay. Briefly, a 10 μL of MTT solution (12 mM) was applied to each well and incubated for 4 h at 37°C. Cells were then lysed in DMSO, and the absorbance of the formazan was measured at 550 nm using a plate reader (Victor 1420 multilabel counter, Perkin Elmer). The results are presented as a fraction of untreated cells, and calculated as a mean of three independent biological replicates with the standard deviation of the mean.

### Analysis of Cell Culture Growth Dynamics Based on the xCELLigence System

HaCaT cells (CLS 300493) were seeded the day before treatment, in 7 technical replicates for each condition, at a density of 1 × 10^4^ per well (according to the manufacturer’s protocol) on E-plate (ACEA Biosciences Inc.). Cells were grown in a standard humidified incubator at 37°C and 5% CO_2_ atmosphere, in xCELLigence RTCA instrument, (ACEA Biosciences Inc.) in DMEM medium as indicated above. Next day, when cells were in their logarithmic phase of growth, the experiment was carried out. To do this, the plates were removed from the xCELLigence device, spent medium removed by aspiration and changed to a medium containing a studied compound (as indicated). After 15 min incubation at room temperature in darkness, the cells were washed twice with PBS and the fresh medium was added. Then, cells were exposed to 514 nm light (irradiance: 23 mW/cm^2^, 22 min), and returned to an xCELLigence device for 70–120 h. The cell index was measured every 15 min and recorded automatically.

### Statistical Analysis

The results of photodynamic inactivation, are presented as the average of at least three independent experiments. Statistical significance was assessed using Student’s *t*-distribution method with the use of Excel software. Three biological replicates of each strain were included separately into the analysis. For comparison of treated clinical isolates groups, ANOVA (Welch test) and RIR Tukey *post hoc* testing were performed using a STATISTICA 10 software (StatSoft Inc. 2011, United States).

## Results

### Photoinactivation of *Pseudomonas aeruginosa* With RB

**FIGURE 1:**
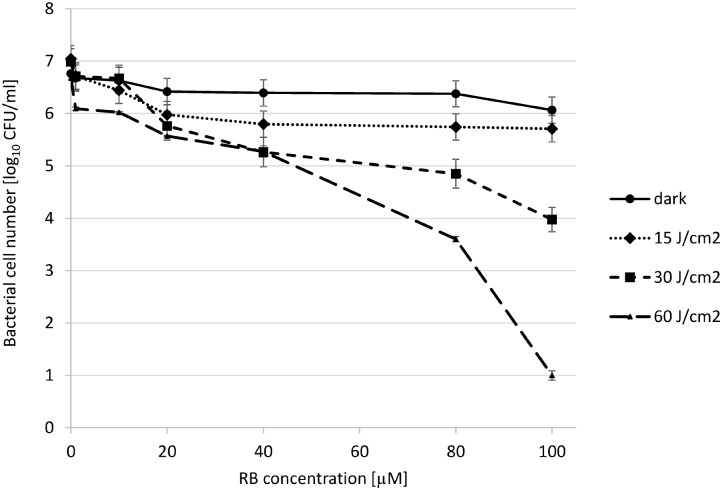
Photoinactivation of *Pseudomonas aeruginosa* 10145 strain with RB. The cells (10^7^ CFU/mL) were incubated with RB at concentrations indicated on the abscissa. Cells were either kept without light application (dark) or they were illuminated with increasing total light doses as indicated in the legend. Each result is a mean ± SE of log_10_ CFU/mL.

In order to assess the initial conditions for photoinactivation of *P. aeruginosa* with the use of RB and 514 nm-light, we subjected the bacterial cells (*P. aeruginosa* 10145 strain) to incubation with increasing concentration of RB, and illuminated cells with various light doses. *P. aeruginosa* is not vulnerable to the action of RB-mediated photokilling at low concentrations of RB (40 μM) and low light doses (15–30 J/cm^2^). We observed that even at a very high concentration of 80 μM RB and a light dose of 30 J/cm^2^, the decrease in the viable cell count was less than 2 log_10_ units. Increasing the RB concentration to a 100 μM resulted in 3 log_10_ units reduction in bacterial count. Accordingly, increasing the light dose to 60 J/cm^2^ resulted in 6 log_10_ units reduction in survival as compared to reference cells (cells not treated with light and RB) (**Figure 1**). It is worth mentioning that the light itself has the impact on survival (Amin et al., 2016) e.g., the 60 J/cm^2^ treatment without RB results in 1 log_10_ units reduction in bacterial count. In further experiments this light dose was excluded, to avoid the influence of light alone on the results of experiments.

### Photoinactivation of *P. aeruginosa* in the Presence of Antimicrobial Peptides Is Increased

**FIGURE 2:**
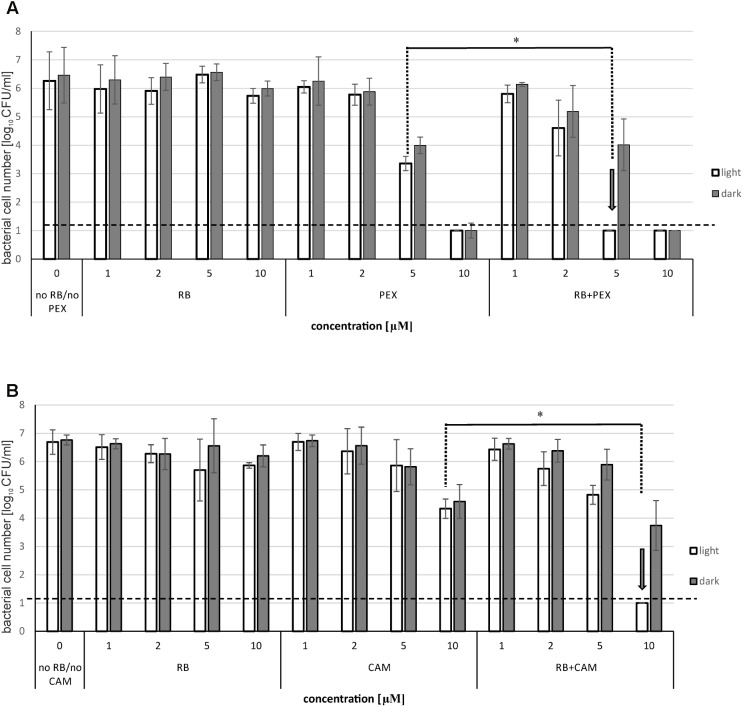
Photoinactivation of *Pseudomonas aeruginosa* with RB in the presence of antimicrobial peptides. The cells were incubated with rose bengal alone (RB), antimicrobial peptide (PEX or CAM) alone, or with rose bengal and one of the antimicrobial peptides together (RB+PEX or RB+CAM). Results for PEX are shown in **(A)**, for CAM in **(B)**. The concentrations of compounds are indicated on the abscissa. In the case of RB and AMP mixtures, cells were either kept without light application (dark) or they were illuminated with a total light dose of 30 J/cm^2^. Each result is a mean ± SE of log_10_ CFU/ml. Significance at respective *p-*values is marked with asterisks (^∗^*p* = 0.002 for PEX, and ^∗^*p* = 0.003 for CAM). Arrows indicate the lowest effective concentration of RB/AMP resulting in a reduction of bacterial cell number below the limit of detection.

Next, we checked the influence of the AMP on the photoinactivation efficiency. To this end we treated the cells with RB and AMP together in a single reaction mixture, incubated for 15 min, and subjected to illumination procedure as described in the Section “Materials and Methods.” We applied RB concentrations up to 10 μM, where no effect on the viability of bacterial cells could be observed. As for the concentrations of CAM and PEX, we chose two different concentrations below MBC value. MBC for PEX was 18 μM, and for CAM – 25 μM. As it can be seen (**Figure 2**), RB itself at concentrations up to 10 μM, does not have any significant impact on *P. aeruginosa* survival, neither in the presence nor in the absence of light. As for the PEX influence on the viability of cells, 5 μM concentration resulted in 2.9 logs reduction, both in the presence and in the absence of light, whereas 10 μM concentration was responsible for the significant decrease in cell number below the limit of detection (**Figure 2A**). The analysis of the combined effect of RB and PEX (5 μM RB and 5 μM PEX) revealed enhanced bactericidal action that resulted in the significant decrease in cell survival (below the limit of detection, >6 logs). A similar trend was observed for the combination of RB with CAM. In this case, the action of 10 μM CAM resulted in 2.08 logs reduction in surviving cells number. When CAM was mixed with RB (both at equal concentrations of 10 μM), and subjected to illumination, the reduction in survival was below the detection limit (**Figure 2B**).

It is worth to notice that the observed significant reduction in the number of viable bacterial cells was connected to the presence of light. This further means that the observed killing was related to a combination of aPDI with AMPs, rather than the interaction of the RB itself with AMP.

### The Presence of Antimicrobial Peptide Affects the Accumulation of RB in *P. aeruginosa* Cells

**FIGURE 3:**
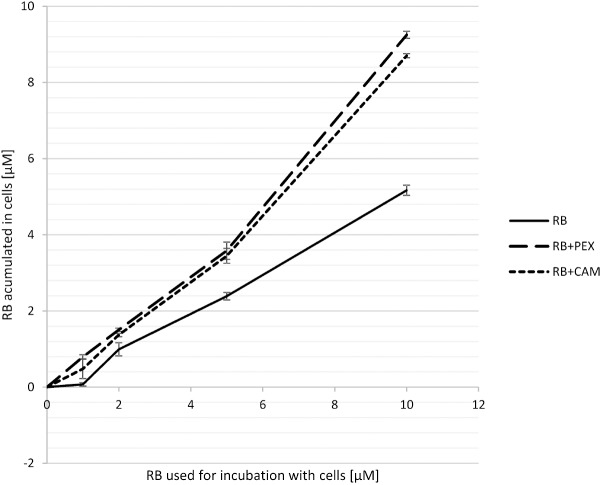
Accumulation of RB in *P. aeruginosa* cells. Cells were incubated for 15 min with RB alone (RB) or with RB in the presence of PEX (RB+PEX) or CAM (RB+CAM). The concentrations of antimicrobial peptides used for incubation was equal to the concentration of RB used (as indicated on the abscissa).

To investigate the basis of the observed enhanced effect of RB and PEX/CAM on bacterial cell survival we analyzed the process of RB accumulation in *P. aeruginosa* cells. Bacterial cells were incubated either with RB alone, or RB and one of the AMPs. After 15 min incubation, cells were pelleted, and the unbound RB remained in the supernatant. The concentration of RB that remained in supernatants was assessed based on the calibration curve and subtracted from RB concentration at which the cells were incubated. In general, when cells were incubated with RB only, the amount of accumulated RB was 5.1 μM (for 10 μM initial RB concentration). The accumulation increased almost twofold for 10 μM initial RB concentration in the presence of either PEX (RB accumulation −9.2 μM) or CAM (RB accumulation −8.7 μM) (**Figure 3**). Interestingly, a similar effect of increased RB accumulation was observed for PEX and for CAM, although the phototoxic effect of aPDI was more pronounced in the presence of RB/PEX than in the presence of RB/CAM (**Figure 2**). No significant difference in RB accumulation efficiency was observed with respect to time the cells were incubated with the studied compounds.

**FIGURE 4:**
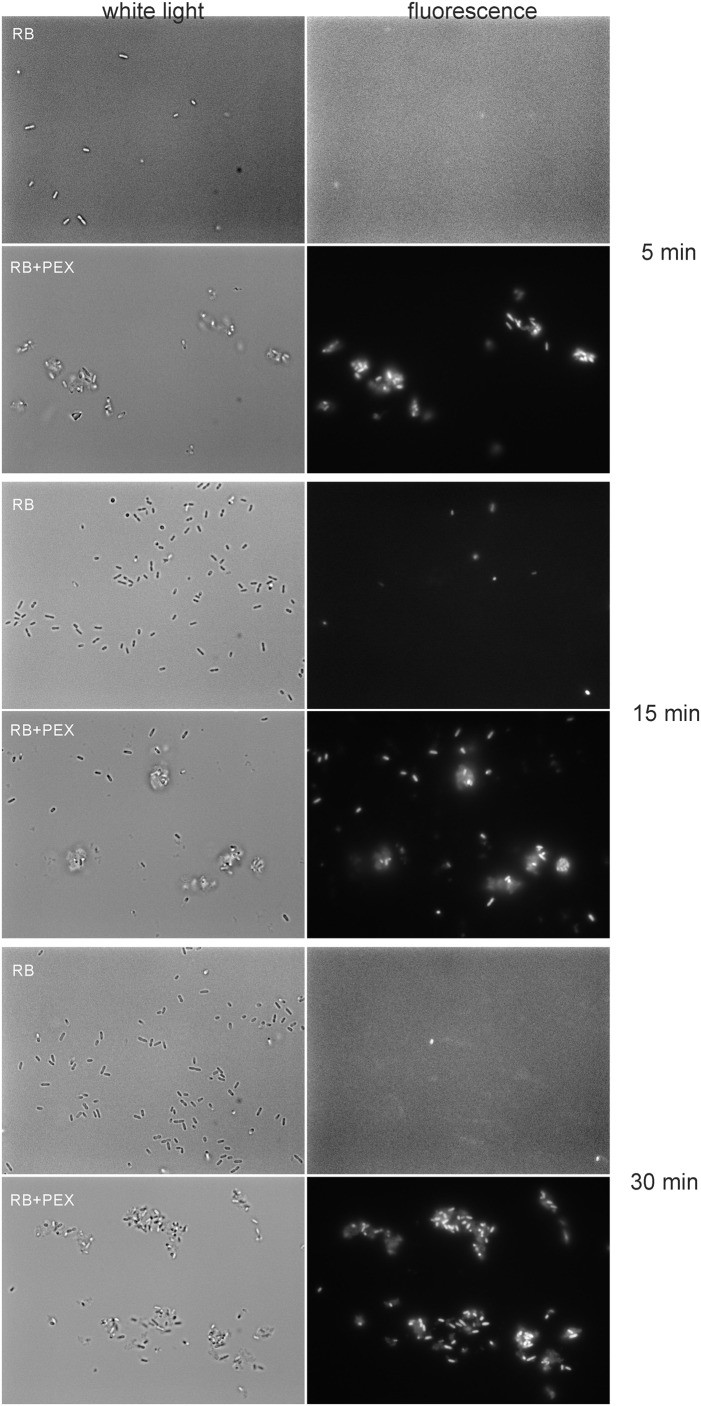
Accumulation of RB in *P. aeruginosa* cells. Cells were incubated with RB (100 μM) or RB+PEX (100 μM each compound) for the time indicated at 37°C. First, the bright field picture was taken, and then immediately fluorescence was observed with the λ_ex_ = 510–550 nm, and λ_em_ > 570 nm.

Accordingly, we observed the enhanced effect of RB accumulation with the use of fluorescence microscopy. To this end, we incubated cells (overnight cultured) in the presence of 100 μM RB. We applied this concentration of RB to obtain sufficient staining of cells. As it can be easily followed, the fluorescence of the cells was not detected in the presence of RB alone, even if the incubation time was prolonged up to 30 min (**Figure 4**). Only upon simultaneous incubation of RB and PEX, the fluorescence of cells could be detected. Moreover, we observed that upon PEX addition the cells had a tendency to group themselves together, however, the observed aggregates should be further studied to indicate their nature. CAM was omitted from the experiments as in tests measuring quantitative accumulation, it presented similar to PEX accumulation profile. In summary, the obtained results indicated that accumulation of RB inside the cells is facilitated upon PEX presence.

### The Integrity of Erythrocytes Is Affected Upon aPDI

**FIGURE 5:**
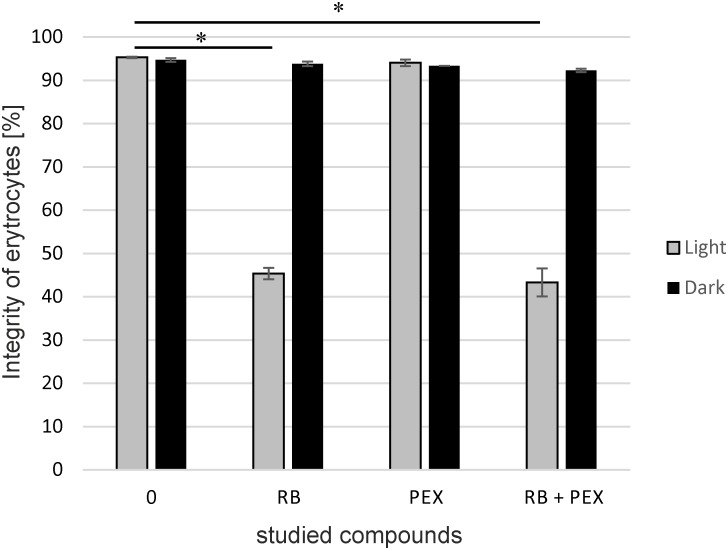
The integrity of erythrocytes membrane. Sheep erythrocytes were incubated with rose bengal (RB), pexiganan (PEX), or both (RB+PEX) at a concentration of 5 μM each, either in darkness (Dark) or upon illumination (Light). Each result is a mean ± SD of a mean. Significance at respective *p-*values is marked with asterisks (^∗^*p* < 0.001 for RB, and RB+PEX with respect to control conditions).

To check biocompatibility of a compound often hemolysis assay is performed as a common measure of safety. Therefore, we checked how the treatment with RB and/or PEX affects the membrane of erythrocytes. The hemolytic behavior of PEX is similar to that of CAM so we omitted the latter from the experiment (Sikora et al., 2018). As it is clearly shown, in dark conditions membranes stay intact for the time of the experiment. We applied 15 min incubation time, the same as for bacterial cell inactivation. Neither presence of RB, PEX nor the mixture of both negatively affects erythrocytes membrane in the experimental conditions in the dark. However, upon light treatment, we observed that about 55% of erythrocytes were subjected to damage. And the process of lysis occurred only when RB and light treatment was performed (**Figure 5**). This indicates that RB and light treatment is phototoxic for erythrocytes. Interestingly, PEX itself did not damage the erythrocytes’ membrane. This further means that PEX is relatively specific toward microorganisms.

### Cell Viability of HaCaT Cells Upon Photodynamic Treatment in the Presence of Antimicrobial Peptides Is Not Disturbed

**FIGURE 6:**
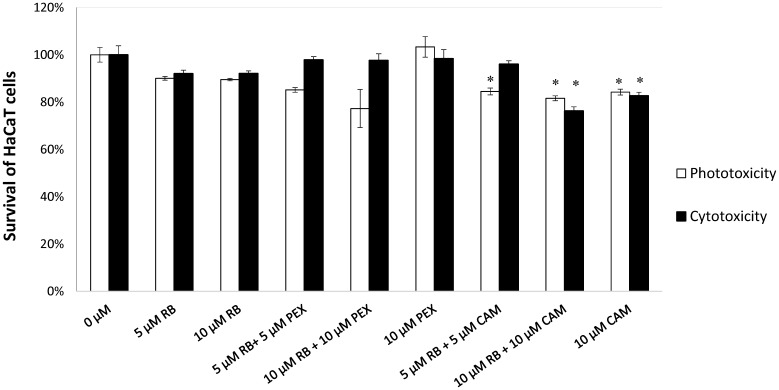
HaCaT cell viability assay. HaCaT cells were treated with compounds indicated on the *X*-axis. Reference cells (0 μM) constitute control conditions where no tested compounds were added. After incubation with particular tested compounds, cells were either subjected to irradiation (23 mW/cm^2^, 30 J/cm^2^) represented by white bars (Phototoxicity) or kept in the dark for the same period of time (gray bars, Cytotoxicity). Each result is a mean ± SD of a mean. Significance at respective *p-*values is marked with asterisks (^∗^*p* < 0.05) with respect to reference cells (0 μM).

To assess the cyto- and phototoxic effect of rose bengal alone or in combination with AMPs on human skin cells the effect of phototreatment on human keratinocytes was measured. We tested the concentrations of the compounds that were used in the bacterial cells inactivation experiments and increased the dose to 10 μM of RB and AMPs. Based on the MTT assay results (**Figure 6**), the viability of the cells was not affected by the presence of RB alone, neither in dark (92.01 and 92.12% survival upon 5 or 10 μM) nor upon irradiation (90.08 and 89.56% survival upon 5 or 10 μM). Also, the combined concentrations of RB and PEX were not cyto- (97.64% survival) nor phototoxic (79.07% survival) even at higher 10 μM concentrations of compounds tested. After incubation of keratinocytes with CAM (10 μM) and upon irradiation, about 15.75% of cells were killed. Accordingly, when cells were incubated with a mixture of CAM and RB (each at 10 μM), 18.36% of cells were photoinactivated. This indicates that mostly CAM was responsible for the observed effect. Similar observations were made after analysis of dark toxicity. Again, CAM alone (10 μM) or in mixture with RB (10 μM) decreased the survival of keratinocytes by 17.3 and 23.73%, respectively. The light itself did not affect membrane integrity. Taken together the results indicate acceptable toxicity for the combined treatment of aPDI with PEX or CAM toward human skin keratinocytes. Although statistical significance was achieved for CAM treatment, the numerical values were not much different from these for PEX (**Supplementary Table S1**). Moreover, ∼80% survival upon treatment is generally considered an acceptable level of toxicity as related to nearly 6 log_10_ reduction in the viable bacterial count. It is also worth to emphasize that PEX itself does not cause HaCaT toxicity (neither with or without light).

As the MTT assay measures the cell viability at the particular time point, we were interested in following the fate of cell growth during the longer time period. To study the eukaryotic cell growth dynamics after phototreatment, we applied the label-free automated monitoring of cell status using micro-electronic sensor plate (E-plate). The change of properties of the analyzed cells influences the passage of electrons and ions on a sensor surface thus providing an information about the biological status of cells. The physical parameter that is measured during such tests is electronic impedance, which is influenced by cell number, viability, morphology, the degree of adhesion. When cells are not present on a sensor E-plate, the impedance of the electrode depends on the ionic environment at the electrodes/solution interface. When the cells are grown on the surface they alter the local ionic environment leading to an increase in electrode impedance. The more cells on the electrodes the larger the change of impedance. The relative change in electrode impedance is represented by Cell Index (CI), which is a quantitative measure of cells attached to the electrodes. The higher the value of CI, the more cells are growing on the plate.

**FIGURE 7:**
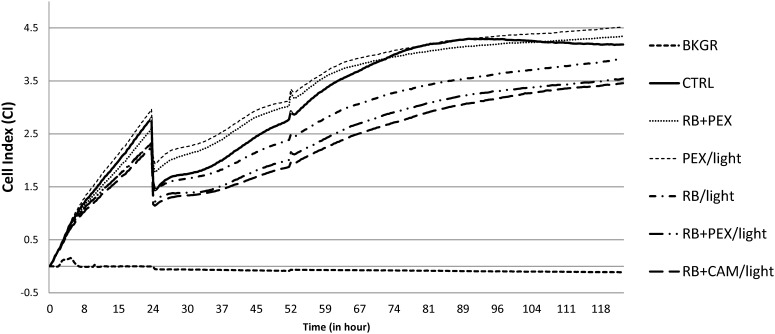
HaCaT cell growth dynamics. Cells were seeded with 10^4^ cells/well, and after the 24 h were treated with an appropriate compound or a combination of compounds. Cell index (CI) was measured every 15 min. An arrow indicates the time of treatment. The presented values are the average of seven replicates. Cells were cultured without any treatment (CTRL), or at logarithmic phase of growth were treated with RB and PEX simultaneously in the dark (RB+PEX), or separately with RB (RB/light) or PEX (PEX/light) in the light. The combinations of photodynamic treatment and antimicrobial peptides was shown as follows: RB and PEX (RB+PEX/light), RB and CAM (RB+CAM/light). The concentrations used were 10 μM for each compound. Cell index in wells containing medium only indicates the background signal (BKGR).

From the experiments based on real-time monitoring of HaCaT cells growth, it can be observed that there is not substantial toxicity or phototoxicity visible upon the treatment. The growth of the cells followed typical for this cell line growth dynamics and during 24 h cells were in exponential phase of growth as indicated by the increasing CI. Cells were incubated outside the incubator for the time of treatment lasting approximately 60 min. After treatment cells resumed growth, and at around 100th-hour control group reached plateau phase. A similar type of behavior was noticed for cells treated with RB and PEX in the dark (**Figure 7** RB+PEX), which indicated that such a mixture was not toxic for keratinocytes. This was also true for PEX only treatment (**Figure 7** PEX/light), in accordance with MTT assay results. A slightly different pattern of growth dynamics was observed for classical photodynamic treatment, namely RB application followed by irradiation (**Figure 7** RB/light). In this case, cell recovery phase was slowed down and the plateau phase was reached about 120th hour of culture. The decrease in growth dynamics was also characteristic for combined treatment of photodynamic action together with PEX (**Figure 7** RB+PEX/light) or CAM (**Figure 7** RB+CAM/light). It should be noticed, however, that cells after the treatment recovered and started to grow, which indicated that the applied treatment was neither cyto- nor phototoxic for human skin keratinocytes.

### Clinical Isolates of *P. aeruginosa* Are Effectively Photoinactivated

As the optimized protocol proved to be effective against reference *P. aeruginosa* 10145 strain, and at the same time safe toward mammalian cells, we were interested if it can be applied against various clinical isolates. As bacterial clinical isolates originating from a particular niche may substantially differ with respect to their properties, we evaluated the combined treatment of aPDI and AMP PEX on several clinical isolates. The analyzed clinical strains were isolated from diverse sources and expressed various antimicrobial resistance pattern (**Table 1**). Within the group of 35 analyzed clinical isolates, there was 14, which were characterized as multidrug- or extended drug-resistant *P. aeruginosa*. Interestingly for some of them, e.g., 156/o, 1885K, 556/k only combined treatment PEX and aPDI was sufficiently effective. All treatments efficiencies for individual test strains are presented in **Table 2**.

**Table 2:** The efficiency of RB-mediated photodynamic inactivation of *P. aeruginosa* clinical isolates in the presence and in the absence of PEX.

Strain		Log_10_ reduction in survival of bacterial cells after treatment with^a^:							
		RB		PEX		RB+PEX		Light only	Antibiotic resistance status^b^
		Light	Dark	Light	Dark	Light	Dark		
1	10145	0.21	0.13	3.34	2.70	5.69	2.68	0.20	
2	3042/s	0.44	−0.12	6.45	6.45	6.45	6.45	0.03	
3	3318/p	0.48	−0.01	5.82	5.82	5.82	5.82	0.14	
4	2969/s	0.35	0.25	6.12	4.49	6.12	6.12	0.20	
5	1561/o	1.11	0.14	1.46	1.33	5.37	2.25	0.42	MDR
6	3404/p	0.81	0.03	5.37	5.37	5.37	5.37	0.36	
7	3117/s	0.35	0.19	6.04	6.04	6.04	6.04	0.30	
8	3146/s	0.63	0.18	1.97	1.48	5.90	2.18	0.43	
9	4190/p A	0.58	0.41	3.44	2.61	6.43	3.07	0.54	MDR
10	1959/o	0.43	0.21	0.98	0.85	5.05	5.05	0.12	XDR
11	3109/o	−0.08	−0.11	5.13	5.13	5.13	5.13	0.01	MDR
12	1286/o	0.36	0.24	0.56	0.55	5.42	2.70	0.19	
13	55/K	0.44	0.24	5.27	5.27	5.27	5.27	0.21	XDR
14	978/K	0.69	−0.08	5.75	2.79	5.75	5.75	0.49	XDR
15	K3	1.10	0.14	5.95	5.95	5.95	5.95	0.02	MDR
16	K4	0.12	0.05	6.04	6.04	6.04	6.04	0.07	XDR
17	792/K	1.16	0.20	5.14	5.14	5.14	5.14	0.37	MDR
18	393/K	1.54	0.19	5.17	2.87	5.17	2.77	0.31	
19	133/K	−0.10	0.00	0.23	0.31	5.02	3.22	0.02	
20	23/K	0.90	0.23	0.67	0.55	5.90	5.90	0.49	
21	887/K	0.68	0.34	5.62	5.62	5.62	5.62	0.49	XDR
22	169/K	2.06	−0.02	2.37	0.52	5.98	5.98	0.07	XDR
23	3752/sz	1.12	0.14	5.85	5.85	5.85	5.85	0.05	
24	4908/p	0.02	−0.37	1.96	1.63	4.88	2.07	0.00	
25	3907/sz	0.92	0.07	3.09	2.37	5.29	3.56	0.86	
26	15/p I	1.12	0.32	5.64	5.64	5.64	5.64	0.16	
27	149/p	0.78	0.44	0.46	0.47	5.97	2.64	0.49	
28	153/s	0.20	0.10	0.49	0.35	6.44	3.38	0.20	
29	547/p	0.37	0.48	2.89	1.64	5.81	4.02	0.14	
30	1885/K	0.79	0.51	1.12	0.83	6.05	2.04	0.09	XDR
31	556/K	0.46	0.29	0.67	0.27	5.41	1.93	0.25	XDR
32	1651/p	0.74	0.22	0.46	−0.28	5.46	2.42	0.03	
33	1304/s	1.25	0.33	5.70	5.70	5.70	5.70	0.65	MDR
34	1804/p	1.08	0.01	5.26	2.60	5.26	2.37	0.25	
35	2284/p	0.87	0.41	1.04	0.97	5.38	2.14	0.09	
36	143/p	0.93	0.50	2.95	2.27	5.83	3.07	0.61	

*^a^The values were calculated by subtracting log_10_ CFU/ml of treated samples from those of untreated controls (0 J/cm^2^; 0 μM RB, 0 μM PEX). The following concentration of compounds was used: 5 μM RB, 5 μM PEX, fluence 30 J/cm^2^. The initial number of cells was ∼10^6^ CFU/ml. The presented values are mean of three independent experiments. ^b^MDR (multidrug resistant), XDR (extended drug resistant), see Section “Materials and Methods” for classification details.*

**FIGURE 8:**
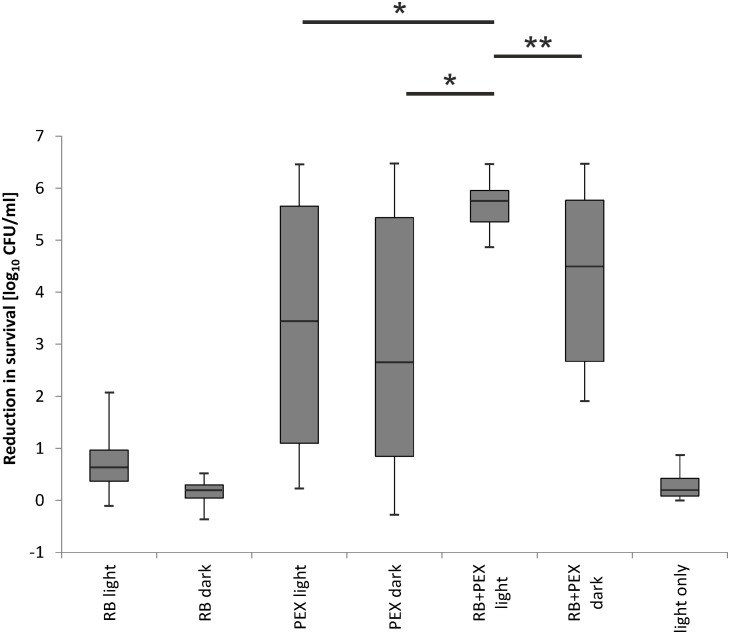
Evaluation of RB-based aPDI treatment combined with antimicrobial peptides against clinical isolates of *P. aeruginosa*. 36 isolates including a reference strain were analyzed with respect to overall aPDI plus PEX treatment. Efficacy of the treatment was assessed in the groups indicated on the abscissa. Each clinical isolate was incubated with RB and subjected to light (RB light) or kept in the dark (RB dark). The results of treatment with PEX upon light exposition (PEX light) and dark toxicity of PEX (PEX dark) are shown. Cells incubated with RB and PEX and irradiated (RB+PEX light), and the same combination kept in the dark (RB+PEX dark) are shown, respectively. Light only treatment is shown as control (light only). All strains were subjected to the same conditions of treatment, i.e., 5 μM of a compound studied, namely RB, PEX. In the case of combined treatment, the concentration of each compound was 5 μM. Irradiance applied was 23 mW/cm^2^, total fluence: 30 J/cm^2^. The error bars represent the minimum and the maximum value of log_10_ unit reduction in viable counts, horizontal lines represent medians. ANOVA with RIR Tukey *post hoc* test analysis was performed to indicate differences between RB+PEX light and the remaining studied groups. Significance at respective *p-*values are marked with asterisks (^∗^*p* = 0.0002, ^∗∗^*p* = 0.004).

We investigated the efficacy of photodynamic inactivation alone, PEX treatment alone, and combined action of both approaches toward all 35 clinical isolates. The experiments were performed with light treatment or in the dark. We observed that in our experimental aPDI protocol photodynamic treatment itself had a very limited influence on cell survival (**Figure 8** RB light). By applying this aPDI protocol, namely 30 J/cm^2^, 5 μM RB we could not observe a significant bactericidal reduction in the number of surviving cells. According to the American Society for Microbiology statement every new approach has to prove an efficacy of min 3 log_10_ reduction of CFU before it can be termed “antimicrobial.” Here, in our research, we indicated the reduction of ≥3 log_10_ units, as antimicrobial reduction that is considered biologically relevant. When cells were treated with PEX only, either with or without the addition of light, the antimicrobial effect could be observed for 55% (20/36) of clinical isolates (**Figure 8** PEX light, PEX dark). In the case of remaining 16 strains, the combination of PEX and RB was sufficient to obtain bactericidal reduction (i.e., reduction ≥3 log_10_ units) even without the addition of light (**Figure 8** PEX+RB dark) for seven isolates. As for the remaining 9 isolates out of the group of 16, the efficient bacterial cells killing (i.e., reduction ≥3 log_10_ units) was only possible after combining PEX with aPDI. Of interest, in this group, the killing efficiency accounted for ≥5 log_10_ units, with a single exception of isolate 4908/p (4.88 log_10_ units reduction). This indicates that only a combination of AMP PEX and photodynamic treatment resulted in the inactivation of all analyzed clinical isolates (**Figure 8** RB+PEX light). Worthy of notice is the fact that after combined treatment the reduction in cell survival was higher than 5 log_10_ units in case of each strain (**Table 2**), again with a single exception already above mentioned.

## Discussion

We showed in our study that inactivation of the Gram-negative bacterium is efficient and can be exploited as potential antimicrobial therapy for treatment of local infections caused by multiresistant *P. aeruginosa*. By the combination of AMP with RB, otherwise inefficient toward Gram-negative bacteria, we obtained a 20-fold decrease in the concentration of RB used in the treatment to obtain min. 5 log_10_ units reduction in cell survival (100 μM/60 J/cm^2^ vs. 5 μM/30 J/cm^2^). This result should be considered as more than a 20-fold decrease as actually lower total fluence was used for the combined treatment as compared to the individual treatment of aPDI (i.e., without AMP). It is worth to emphasize that application of lower fluence (in this case 30 J/cm^2^ vs. 60 J/cm^2^) results also in shortening of irradiation time (in this case 1335 s vs. 2668 s), which is of great clinical importance. The enhanced photokilling effect of the combined action of aPDI and AMP was due to the facilitated accumulation of RB in bacterial cells in the presence of AMP as compared to cells incubated only with RB (**Figures 3**, **4**). Recently it was shown that in the presence of AMP aurein 1.2, the uptake of methylene blue (MB) was twice as much as compared to MB uptake alone. This doubled value of uptake was observed also in presented work for a combination of RB and CAM or RB and PEX (**Figure 3**). It is known from the literature data that the uptake process of a PS is not the only and critical determinant of efficient photodynamic inactivation. This further means that a PS does not have to be accumulated inside the cells to perform an efficient photodynamic action (Preuss et al., 2013). For example, chlorin-e6 uptake was significantly decreased in the presence of AMP, however, the synergistic action of aPDI and AMP was still observed (de Freitas et al., 2018). Such a characteristic of aPDI is an advantage as it means that bactericidal action can be started from the outside of the cell rather than from the inside where DNA could be affected and potentially mutagenized. We did not observe any difference in facilitating RB uptake between the two AMPs applied. Both CAM and PEX peptides had a similar impact on the accumulation of RB in *P. aeruginosa* cells. These two peptides have similar properties as for their structural features (helical peptides), and net positive charge (+6, and +10 for CAM and PEX, respectively), however, PEX was more potent in exhibiting antimicrobial action. PEX concentration reduced by 50% was sufficient to obtain a similar bactericidal effect as compared to CAM.

When considering the application of combined action of aPDI and AMPs, both components should be taken into account, namely PS and AMP. From the previously published research, it is known that not all combination of aPDI an AMP are robust and can be effective. Aurein 1.2 was effective when combined with aPDI, but only when MB or chlorin e6 were used as photosensitizers. In the case of another photosensitizer curcumin, the synergistic effect was not observed at all. This proves that the enhanced action of aPDI and AMP was PS-dependent (de Freitas et al., 2018). In our studies, RB proved to be very good PS for combined aPDI and AMP action, whereas it is commonly known that RB itself is not effective against Gram-negative species (Bezman et al., 1978; Schafer et al., 2000; Demidova and Hamblin, 2005; Wen et al., 2017). This photosensitizer efficiently produces singlet oxygen, however, the outer membrane present in Gram-negatives prevents anionic RB from reaching the critical cellular structures like a cytoplasmic membrane. Thus, our hypothesis to employ cationic AMP that increases outer membrane permeability and allows for the efficient accumulation of RB in cells proved valid. Our results from accumulation experiments confirmed the rationale of our hypothesis. It could be of further interest to explore if AMP facilitates RB loading into cells via direct interaction between the two, or AMP changes the properties of the cellular membrane, which further results in more efficient accumulation of RB. While the process of PS accumulation is a light-independent phenomenon, i.e., no light is necessary for the process to occur, aPDI is from the definition a light-dependent process. The significant reduction in the number of viable bacterial cells was observed in the presence of light. This further means that the observed increased killing was related to a combination of aPDI (RB+light) with AMPs, rather than the interaction of the RB itself with AMP, at least for the particular concentrations tested. On the other hand, we could observe that application of RB together with PEX in dark resulted in enhanced bactericidal activity for about 30% of isolates. This observation points that RB itself facilitates PEX action, perhaps as a result of direct interaction. Further research is needed to explore the nature of the interaction between AMPs and RB.

The concept of improving the uptake of a PS in bacterial cells due to the action of the AMP, or more broadly cationic peptide, has been studied and described. Within the last few years, the results on the activity of porphyrin or xanthenes combined with cationic peptides against the model Gram-positive (*S. aureus*) and Gram-negative (mostly *Escherichia coli*, *P. aeruginosa*) species have been described (Bourre et al., 2010; Yang et al., 2011; Liu et al., 2012; Dosselli et al., 2013, 2014; Johnson et al., 2013; Costley et al., 2017; Le Guern et al., 2017, 2018). In all the referenced work, however, the authors presented the construction of conjugates composed of a PS and AMP rather than mixing separate components. The authors applied similar fluences as these presented in the current study, i.e., from 10 to 30 J/cm^2^, and concentrations of 1 μM–1 mM to obtain at least 3–4 log_10_ reduction in *P. aeruginosa* cell survival. Only in a single study the same photosensitizer as in the presented study, namely RB, was used. RB-(KLAKLAK)_2_ conjugate activity was presented against *P. aeruginosa*, indicating 2.5 log_10_ reduction upon 10 μM concentration (Costley et al., 2017). The same peptide was conjugated to, structurally similar to RB, eosin producing finally a conjugate that at 10 μM concentration resulted in >5 log_10_ reduction in cell survival. However, in this case much higher fluence of 250 J/cm^2^ was applied (Johnson et al., 2013). In our study, low light doses and low RB and AMPs concentrations resulted in the efficient killing of *P. aeruginosa* without conjugation of the two elements. In the only study concerning the evaluation of mixtures of PS (MB, chlorin e6, curcumin) and AMP (aurein 1.2), the applied combinations were not as much effective toward Gram-negative species (*A. baumannii*, *E. coli*). In contrast, our combinations were effective against Gram-negative species, and even toward diverse clinical isolates.

For any antimicrobial compound, no appreciable toxicity toward mammalian cells should be noted. For the mentioned eosin-(KLAKLAK)_2_ 50% toxicity toward HaCaT cells was noticed (Johnson et al., 2013). This was in contrast to our results, where only limited up to 20% cyto- and phototoxicity was observed. Moreover, in our presented work, we showed for the first time that the growth dynamics of HaCaT cells after photodynamic treatment and/or combined treatment was not significantly disturbed. Our studies were the first to show, in contrast to the so far applied single time-point measurements, the dynamics of cell proliferation for several days. We could also observe a good agreement of real-time monitoring of growth dynamics with commonly applied MTT assay. In some cases, however, obtaining sufficient range of “therapeutic window,” i.e., substantial reduction of bacterial cell number, while sparing host cells could not be obtained at all, although the obtained conjugates were very potent with respect to their antimicrobial properties (Le Guern et al., 2017). Summarizing, based on the results obtained by combining aPDI with AMPs is a viable approach to obtain efficient killing of bacterial cells, and preserve no toxicity against human cells. Moreover, we believe that in particular cases, including the example presented within our research, simple mixing of PS and AMP is sufficient to obtain substantial antimicrobial activity, without the need of laborious conjugation process. At the same time, we have observed that RB+light treatment was hemolytic in our experimental *in vitro* conditions. We could see 55% hemolysis, which was a statistically significant value. The question remains about biological meaning of this observation in terms of the potential application of aPDI in clinics. From the definition, aPDI is a localized process, and the photosensitizer is applied topically, thus it actually has little impact on blood components degradation. Interestingly, there are several excellent works published by Kochevar group on the application of RB with green light to assess *in vivo* (Dutch belted rabbits model) the potential damage of retina and iris, and the treatment was assessed safe, rapid and effective (Zhu et al., 2016; Gallego-Munoz et al., 2017). Retina from RB+light-treated eyes seemed normal and choriocapillaris contained intact erythrocytes (Zhu et al., 2016).

All the so far research concerning the discussed subject were performed on a very limited number of isolates, whereas it is evident that due to the huge genotypic and phenotypic variation of bacterial isolates, evaluating the results on many strains is of real value and actual necessity. Here, we evaluated 35 clinical isolates, which originate from various sources to verify if the combined protocol of aPDI and PEX treatment can be applied to a wide repertoire of strains. In our experimental conditions all strains could be eradicated, i.e., the survival of bacterial cells dropped below the limit of detection. To evaluate the robustness of our protocol against clinical strains, we applied conditions optimized for reference *P. aeruginosa* 10145 strain. This particular strain was vulnerable to the action of PEX and for the combination of PEX and RB in the dark, and upon light activation for 5 μM PEX and 5 μM RB combination. Based on the results obtained for reference strain, we employed the same concentrations and fluence (30 J/cm^2^) for the analysis of all the clinical isolates. As it appeared for almost half of the analyzed strains (47%) mere presence of PEX was sufficient to effectively reduce the number of bacterial cells >5 log_10_ units. This means that PEX MBC values for these strains were much lower as compared to the reference 10145 strain. As a result, the concentration of PEX used for these experiments could be further reduced.

As aPDI practically from the definition is a localized treatment the most interesting strains are those isolated from local infections, e.g., wound, ear or bronchi. From the clinical point of view, the most important parameter of bacterial characterization is antibiotic resistance profile. In the previous work published by our group and others, it was shown that aPDI is equally effective toward antibiotic resistant and antibiotic susceptible strains (Maisch et al., 2014; Makdoumi and Backman, 2016). Here, the same applies to the combined treatment of aPDI and AMPs. Even strains that were considered extended drug-resistant were efficiently photoinactivated. As the WHO pathogens list includes carbapenem-resistant *P. aeruginosa*, we included such isolates into the analysis, proving the robustness of our approach also on these critical pathogens (**Tables 1**, **2**).

We chose RB as PS for our studies due to its photophysical properties, like the high production of singlet oxygen. Production of singlet oxygen in antimicrobial photodynamic therapy is highly appreciated as there are no effective mechanisms that could protect bacterial cells from the toxic singlet oxygen action. Moreover, RB is commonly used in various medical applications. This pink xanthene dye is used for the diagnosis of damage in corneal (Doughty, 2013), brucellosis (Ducrotoy and Bardosh, 2017). Rose Bengal in combination with the green light has been extensively studied with respect to photochemical tissue bonding and corneal transplants (Gu et al., 2011; Gallego-Munoz et al., 2017). This compound was also studied in anti-tumor immune responses in melanoma models (Liu et al., 2018). In terms of aPDI, RB was underappreciated due to its poor action against Gram-negative species. However, our results as well recent from other groups (Wen et al., 2017; Li et al., 2018) proved that RB is efficient photosensitizer also toward Gram-negative bacteria.

Methylene blue was more efficiently accumulated in *Enterococcus faecalis* cells upon the presence of the peptide aurein 1.2, thus delivering a higher local concentration of ROS produced upon light treatment. In the case of chlorin Ce6, however, direct interaction with AMP was postulated through hydrophobic interactions, which resulted in the prolonged half-life of the PS, thus providing an efficient enhancement of aPDI (de Freitas et al., 2018). At this stage, we are not able to identify the exact mechanism of enhanced bactericidal action of RB-based aPDI combined with PEX or CAM. We can only speculate about possible explanations, which can be ascribed to the improved action of either AMP or PS or interactions between the two, which might influence the structure or the function of each molecule. In our studied combination of RB+PEX/CAM the observed enhanced antimicrobial effect might be related to RB and AMP direct interaction, as we observed efficient killing of bacterial cells, not only in the presence of light but also in its absence (**Table 2**). RB seems to interact with PEX/CAM directly. The analysis of UV-Vis absorption spectra of RB alone and in combination with AMPs (less intense, red-shifted maximum absorption peak) might confirm such hypothesis (**Supplementary Figure S1**). The overall picture seems to be more complex as a yet higher increase in antimicrobial efficacy of RB combined with AMPs is observed upon light irradiation. So overall the hypothesis explaining the enhanced action of aPDI and AMP studied by us can be proposed. Based on this hypothesis direct interaction of RB and AMPs occurs with no light share, which results in (i) higher accumulation of RB in bacterial cells, (ii) change of function and/or structure of PEX into a more potent membrane-disorganizing agent. In addition, upon light treatment, production of ROS occurs, which further increases the observed bactericidal effect.

When new approaches are introduced as potential medical applications, toxicity and biocompatibility issues should be investigated. In our experimental conditions, AMPs were not toxic at the concentrations tested (up to 10 μM). The combined action of AMP and aPDI showed, however, some toxicity (50% in hemolysis assay, 20% MTT assay). Such toxicity is acceptable, particularly if the treatment has local character. At the same time, the observation of HaCaT cell growth for a longer time revealed that cells after treatment took up growth and proliferation occurred. The dynamics was retarded in relation to untreated cells, but without any cytostatic effect observed. This indicates that the studied treatment has the potential to be exploited as a rational clinical application. More studies on complex *in vivo* models are required to eventually verify this hypothesis.

## Conclusion

We proved that combination of RB-based antimicrobial photodynamic (aPDI) inactivation and AMPs is a viable alternative to classical antimicrobials, effective against multidrug-resistant and extended drug-resistant *P. aeruginosa*. This is the first study showing the significant effect of the combined action of aPDI and AMPs toward 35 clinical isolates with diverse antimicrobial resistance, resulting in equally effective photokilling. Specifically, AMP pexiganan (PEX) was found to be a very potent molecule with evident selectivity toward Gram-negative bacterial cells. Clever and careful combination of photodynamic inactivation and AMPs opens up new possibilities for combating pathogens that cause localized infections. This proposed approach could help to design strategies that alleviate the increasingly common problem of antimicrobial resistance spreading.

## Author Contributions

JN conceived the study, analyzed the results and wrote the first draft of the manuscript, and performed some experiments. KW, PO, DN, and AB carried out experiments. WK provided technical support and helped to draft the manuscript. All authors contributed to manuscript revision, read and approved the submitted version.

## Conflict of Interest Statement

The authors declare that the research was conducted in the absence of any commercial or financial relationships that could be construed as a potential conflict of interest.

## Supplementary Material

FIGURE S1 UV-Vis spectra of RB alone and RB in the presence of antimicrobial peptides CAMEL and pexiganan. Light absorption spectra were of analyzed compounds at concentration of 2 μM were measured in a wavelength range of 450–650 nm with 0.5 nm intervals, in quartz cuvettes (1 cm light path) containing appropriate solutions in 1 mL PBS, pH 6.8, using Beckman’s DU 650 spectrophotometer at room temperature (25°C).

TABLE S1 Numerical values of HaCaT cell viability assay (MTT assay) results are presented in **Figure 6**.

## References

[B1] Amin R. M. Bhayana B. Hamblin M. R. Dai T. (2016). Antimicrobial blue light inactivation of *Pseudomonas aeruginosa* by photo-excitation of endogenous porphyrins: In vitro and in vivo studies. *Lasers Surg. Med.* 48 562–568. 10.1002/lsm.22474 26891084 PMC4914480

[B2] Bertoloni G. Rossi F. Valduga G. Jori G. Van L. J. (1990). Photosensitizing activity of water- and lipid-soluble phthalocyanines on *Escherichia coli*. *FEMS Microbiol. Lett.* 59 149–155. 10.1111/j.1574-6968.1990.tb03814.x 2125956

[B3] Bezman S. A. Burtis P. A. Izod T. P. Thayer M. A. (1978). Photodynamic inactivation of *E. coli* by rose bengal immobilized on polystyrene beads. *Photochem. Photobiol.* 28 325–329. 10.1111/j.1751-1097.1978.tb07714.x 360250

[B4] Blanc D. S. Nahimana I. Petignat C. Wenger A. Bille J. Francioli P. (2004). Faucets as a reservoir of endemic *Pseudomonas aeruginosa* colonization/infections in intensive care units. *Intensive Care Med.* 30 1964–1968. 10.1007/s00134-004-2389-z 15257431

[B5] Bourre L. Giuntini F. Eggleston I. M. Mosse C. A. Macrobert A. J. Wilson M. (2010). Effective photoinactivation of Gram-positive and Gram-negative bacterial strains using an HIV-1 Tat peptide-porphyrin conjugate. *Photochem. Photobiol. Sci.* 9 1613–1620. 10.1039/c0pp00146e 20931134

[B6] Brown E. D. Wright G. D. (2016). Antibacterial drug discovery in the resistance era. *Nature* 529 336–343. 10.1038/nature17042 26791724

[B7] Ceniceros A. Pertega S. Galeiras R. Mourelo M. Lopez E. Broullon J. (2016). Predicting mortality in burn patients with bacteraemia. *Infection* 44 215–222. 10.1007/s15010-015-0847-x 26449237

[B8] CLSI (2012). *Performance Standards for Antimicrobial Susceptibility Testing. CLSI Approved Standard* M100-S22. Wayne, PA: Clinical and Laboratory Standards Institute.

[B9] Cockerill F. R. Wikler M. A. Alder J. Dudley M. N. Eliopoulos G. M. Ferraro M. J. (2012). *Methods for Dilution Antimicrobial Susceptibility Tests for Bacteria that Grow Aerobically; Approved Standards*, 8th Edn. Wayne, PA: CLSI.

[B10] Costley D. Nesbitt H. Ternan N. Dooley J. Huang Y. Y. Hamblin M. R. (2017). Sonodynamic inactivation of Gram-positive and Gram-negative bacteria using a Rose Bengal-antimicrobial peptide conjugate. *Int. J. Antimicrob. Agents* 49 31–36. 10.1016/j.ijantimicag.2016.09.034 27908581 PMC5191983

[B11] D’Agata E. (2014). *Pseudomonas aeruginosa* and other *Pseudomonas* Species. Philadelphia, PA: Elsevier.

[B12] de Freitas L. M. Lorenzon E. N. Santos-Filho N. A. Zago L. H. P. Uliana M. P. De Oliveira K. T. (2018). Antimicrobial Photodynamic therapy enhanced by the peptide aurein 1.2. *Sci. Rep.* 8:4212. 10.1038/s41598-018-22687-x 29523862 PMC5844988

[B13] Demidova T. N. Hamblin M. R. (2005). Effect of cell-photosensitizer binding and cell density on microbial photoinactivation. *Antimicrob. Agents Chemother.* 49 2329–2335. 10.1128/AAC.49.6.2329-2335.2005 15917529 PMC1140487

[B14] Dosselli R. Ruiz-Gonzalez R. Moret F. Agnolon V. Compagnin C. Mognato M. (2014). Synthesis, spectroscopic, and photophysical characterization and photosensitizing activity toward prokaryotic and eukaryotic cells of porphyrin-magainin and -buforin conjugates. *J. Med. Chem.* 57 1403–1415. 10.1021/jm401653r 24456407

[B15] Dosselli R. Tampieri C. Ruiz-Gonzalez R. De Munari S. Ragas X. Sanchez-Garcia D. (2013). Synthesis, characterization, and photoinduced antibacterial activity of porphyrin-type photosensitizers conjugated to the antimicrobial peptide apidaecin 1b. *J. Med. Chem.* 56 1052–1063. 10.1021/jm301509n 23231466

[B16] Doughty M. J. (2013). Rose bengal staining as an assessment of ocular surface damage and recovery in dry eye disease-a review. *Cont. Lens Anterior Eye* 36 272–280. 10.1016/j.clae.2013.07.008 23928365

[B17] Ducrotoy M. J. Bardosh K. L. (2017). How do you get the Rose Bengal Test at the point-of-care to diagnose brucellosis in Africa? The importance of a systems approach. *Acta Trop.* 165 33–39. 10.1016/j.actatropica.2016.10.004 27725154

[B18] Fila G. Kasimova K. Arenas Y. Nakonieczna J. Grinholc M. Bielawski K. P. (2016). Murine Model Imitating Chronic wound infections for evaluation of antimicrobial photodynamic therapy efficacy. *Front. Microbiol.* 7:1258. 10.3389/fmicb.2016.01258 27555843 PMC4977341

[B19] Fila G. Kawiak A. Grinholc M. S. (2017). Blue light treatment of *Pseudomonas aeruginosa*: strong bactericidal activity, synergism with antibiotics and inactivation of virulence factors. *Virulence* 8 938–958. 10.1080/21505594.2016.1250995 27763824 PMC5626244

[B20] Gallego-Munoz P. Ibares-Frias L. Lorenzo E. Marcos S. Perez-Merino P. Bekesi N. (2017). Corneal wound repair after rose bengal and green light crosslinking: clinical and histologic Study. *Invest. Ophthalmol. Vis. Sci.* 58 3471–3480. 10.1167/iovs.16-21365 28700779

[B21] Giuliani F. Martinelli M. Cocchi A. Arbia D. Fantetti L. Roncucci G. (2010). In vitro resistance selection studies of RLP068/Cl, a new Zn(II) phthalocyanine suitable for antimicrobial photodynamic therapy. *Antimicrob. Agents Chemother* 54 637–642. 10.1128/AAC.00603-09 20008782 PMC2812146

[B22] Gu C. Ni T. Verter E. E. Redmond R. W. Kochevar I. E. Yao M. (2011). Photochemical tissue bonding: a potential strategy for treating limbal stem cell deficiency. *Lasers Surg. Med.* 43 433–442. 10.1002/lsm.21066 21674548

[B23] Hamblin M. R. O’donnell D. A. Murthy N. Rajagopalan K. Michaud N. Sherwood M. E. (2002). Polycationic photosensitizer conjugates: effects of chain length and Gram classification on the photodynamic inactivation of bacteria. *J. Antimicrob. Chemother* 49 941–951. 10.1093/jac/dkf053 12039886

[B24] Hashimoto M. C. Prates R. A. Kato I. T. Nunez S. C. Courrol L. C. Ribeiro M. S. (2012). Antimicrobial Photodynamic Therapy on Drug-resistant *Pseudomonas aeruginosa*-induced Infection. An In Vivo Study(dagger). *Photochem. Photobiol* 88 590–595. 10.1111/j.1751-1097.2012.01137.x 22404212

[B25] Johnson G. A. Muthukrishnan N. Pellois J. P. (2013). Photoinactivation of Gram positive and Gram negative bacteria with the antimicrobial peptide (KLAKLAK)(2) conjugated to the hydrophilic photosensitizer eosin Y. *Bioconjug. Chem.* 24 114–123. 10.1021/bc3005254 23240991

[B26] Lautenbach E. Synnestvedt M. Weiner M. G. Bilker W. B. Vo L. Schein J. (2010). Imipenem resistance in *Pseudomonas aeruginosa*: emergence, epidemiology, and impact on clinical and economic outcomes. *Infect. Control Hosp. Epidemiol.* 31 47–53. 10.1086/649021 19951202

[B27] Le Guern F. Ouk T. S. Ouk C. Vanderesse R. Champavier Y. Pinault E. (2018). Lysine analogue of polymyxin B as a significant opportunity for photodynamic antimicrobial chemotherapy. *ACS Med. Chem. Lett.* 9 11–16. 10.1021/acsmedchemlett.7b00360 29348804 PMC5767886

[B28] Le Guern F. Sol V. Ouk C. Arnoux P. Frochot C. Ouk T. S. (2017). Enhanced photobactericidal and targeting properties of a cationic porphyrin following the attachment of polymyxin B. *Bioconjug. Chem.* 28 2493–2506. 10.1021/acs.bioconjchem.7b00516 28853858

[B29] Li C. Lin F. Sun W. Wu F. G. Yang H. Lv R. (2018). Self-Assembled rose bengal-exopolysaccharide nanoparticles for improved photodynamic inactivation of bacteria by enhancing singlet oxygen generation directly in the solution. *ACS Appl. Mater Interfaces* 10 16715–16722. 10.1021/acsami.8b01545 29641169

[B30] Liu F. Soh Yan Ni A. Lim Y. Mohanram H. Bhattacharjya S. Xing B. (2012). Lipopolysaccharide neutralizing peptide-porphyrin conjugates for effective photoinactivation and intracellular imaging of gram-negative bacteria strains. *Bioconjug. Chem.* 23 1639–1647. 10.1021/bc300203d 22769015

[B31] Liu H. Weber A. Morse J. Kodumudi K. Scott E. Mullinax J. (2018). T cell mediated immunity after combination therapy with intralesional PV-10 and blockade of the PD-1/PD-L1 pathway in a murine melanoma model. *PLoS One* 13:e0196033. 10.1371/journal.pone.0196033 29694419 PMC5918896

[B32] Ludvikova L. Fris P. Heger D. Sebej P. Wirz J. Klan P. (2016). Photochemistry of rose bengal in water and acetonitrile: a comprehensive kinetic analysis. *Phys Chem Chem Phys* 18 16266–16273. 10.1039/c6cp01710j 27253480

[B33] Magiorakos A. P. Srinivasan A. Carey R. B. Carmeli Y. Falagas M. E. Giske C. G. (2012). Multidrug-resistant, extensively drug-resistant and pandrug-resistant bacteria: an international expert proposal for interim standard definitions for acquired resistance. *Clin. Microbiol. Infect.* 18 268–281. 10.1111/j.1469-0691.2011.03570.x 21793988

[B34] Maisch T. Eichner A. Spath A. Gollmer A. Konig B. Regensburger J. (2014). Fast and effective photodynamic inactivation of multiresistant bacteria by cationic riboflavin derivatives. *PLoS One* 9:e111792. 10.1371/journal.pone.0111792 25469700 PMC4254278

[B35] Makdoumi K. Backman A. (2016). Photodynamic UVA-riboflavin bacterial elimination in antibiotic-resistant bacteria. *Clin. Exp. Ophthalmol.* 44 582–586. 10.1111/ceo.12723 26867998

[B36] Nitzan Y. Gutterman M. Malik Z. Ehrenberg B. (1992). Inactivation of gram-negative bacteria by photosensitized porphyrins. *Photochem. Photobiol* 55 89–96. 10.1111/j.1751-1097.1992.tb04213.x 1534909

[B37] Ogonowska P. Wozniak A. Pieranski M. K. Wasylew T. Kwiek P. Brasel M. (2018). Application and characterization of new light-emitting diodes for photodynamic inactivation. *Light. Res. Technol.* 1–13.

[B38] Potron A. Poirel L. Nordmann P. (2015). Emerging broad-spectrum resistance in *Pseudomonas aeruginosa* and *Acinetobacter baumannii*: mechanisms and epidemiology. *Int. J. Antimicrob. Agents* 45 568–585. 10.1016/j.ijantimicag.2015.03.001 25857949

[B39] Preuss A. Zeugner L. Hackbarth S. Faustino M. A. Neves M. G. Cavaleiro J. A. (2013). Photoinactivation of *Escherichia coli* (SURE2) without intracellular uptake of the photosensitizer. *J. Appl. Microbiol.* 114 36–43. 10.1111/jam.12018 22978364

[B40] Rice L. B. (2008). Federal funding for the study of antimicrobial resistance in nosocomial pathogens: no ESKAPE. *J. Infect. Dis.* 197 1079–1081. 10.1086/533452 18419525

[B41] Schafer M. Schmitz C. Facius R. Horneck G. Milow B. Funken K. H. (2000). Systematic study of parameters influencing the action of Rose Bengal with visible light on bacterial cells: comparison between the biological effect and singlet-oxygen production. *Photochem. Photobiol.* 71 514–523. 10.1562/0031-8655(2000)071<0514:SSOPIT>2.0.CO;2 10818781

[B42] Sikora K. Jaskiewicz M. Neubauer D. Bauer M. Bartoszewska S. Baranska-Rybak W. (2018). Counter-ion effect on antistaphylococcal activity and cytotoxicity of selected antimicrobial peptides. *Amino Acids* 50 609–619. 10.1007/s00726-017-2536-9 29307075 PMC5917001

[B43] Tavares A. Carvalho C. M. Faustino M. A. Neves M. G. Tome J. P. Tome A. C. (2010). Antimicrobial photodynamic therapy: study of bacterial recovery viability and potential development of resistance after treatment. *Mar. Drugs* 8 91–105. 10.3390/md8010091 20161973 PMC2817925

[B44] Tegos G. P. Anbe M. Yang C. Demidova T. N. Satti M. Mroz P. (2006). Protease-stable polycationic photosensitizer conjugates between polyethyleneimine and chlorin(e6) for broad-spectrum antimicrobial photoinactivation. *Antimicrob. Agents Chemother.* 50 1402–1410. 10.1128/AAC.50.4.1402-1410.2006 16569858 PMC1426948

[B45] Tsuchikama K. Shimamoto Y. Anami Y. (2017). Truncated Autoinducing Peptide Conjugates Selectively Recognize and Kill Staphylococcus aureus. *ACS Infect Dis* 3 406–410. 10.1021/acsinfecdis.7b00013 28155275

[B46] Wainwright M. Maisch T. Nonell S. Plaetzer K. Almeida A. Tegos G. P. (2017). Photoantimicrobials-are we afraid of the light? *Lancet Infect Dis* 17 e49–e55. 10.1016/S1473-3099(16)30268-7 27884621 PMC5280084

[B47] Wen X. Zhang X. Szewczyk G. El-Hussein A. Huang Y. Y. Sarna T. (2017). Potassium iodide potentiates antimicrobial photodynamic inactivation mediated by rose bengal in in vitro and in vivo studies. *Antimicrob Agents Chemother.* 61 e467–e417. 10.1128/AAC.00467-17 28438946 PMC5487662

[B48] Yang K. Gitter B. Ruger R. Wieland G. D. Chen M. Liu X. (2011). Antimicrobial peptide-modified liposomes for bacteria targeted delivery of temoporfin in photodynamic antimicrobial chemotherapy. *Photochem. Photobiol. Sci.* 10 1593–1601. 10.1039/c1pp05100h 21773628

[B49] Zasloff M. (2002). Antimicrobial peptides of multicellular organisms. *Nature* 415 389–395. 10.1038/415389a 11807545

[B50] Zhu H. Alt C. Webb R. H. Melki S. Kochevar I. E. (2016). Corneal crosslinking with rose bengal and green light: efficacy and safety evaluation. *Cornea* 35 1234–1241. 10.1097/ICO.0000000000000916 27362877

