# Supplementary material for: Rose Bengal-Mediated Photoinactivation of Multidrug Resistant Pseudomonas aeruginosa Is Enhanced in the Presence of Antimicrobial Peptides
Source: Front Microbiol. 2018 Aug 20;9:1949. doi: 10.3389/fmicb.2018.01949 (PMC6110182; doi:10.3389/fmicb.2018.01949)
Supplement: FIGURE S1 — UV-Vis spectra of RB alone and RB in the presence of antimicrobial peptides CAMEL and pexiganan. Light absorption spectra were of analyzed compounds at concentration of 2 μM were measured in a wavelength range of 450–650 nm with 0.5 nm intervals, in quartz cuvettes (1 cm light path) containing appropriate solutions in 1 mL PBS, pH 6.8, using Beckman’s DU 650 spectrophotometer at room temperature (25°C). [file Image_1.PDF]

Supplementary Figure 1

Rose Bengal-mediated photoinactivation of multidrug resistant *Pseudomonas aeruginosa* is enhanced in the presence of antimicrobial peptides

Joanna Nakonieczna*, Katarzyna Wolnikowska, Patrycja Ogonowska, Damian Neubauer, Agnieszka Bernat, Wojciech Kamysz

*** Correspondence:** joanna.nakonieczna@biotech.ug.edu.pl

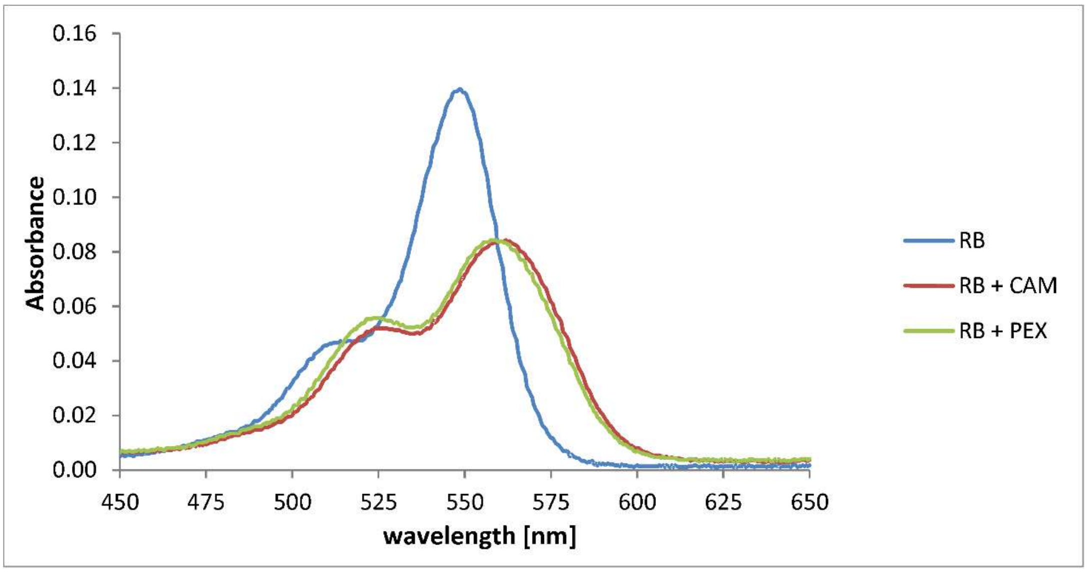

Supplementary Figure 2. UV-Vis spectra of RB alone and RB in the presence of antimicrobial peptides CAMEL and pexiganan.

Light absorption spectra were of analyzed compounds at concentration of 2 µM were measured in a wavelength range of 450 – 650 nm with 0.5 nm intervals, in quartz cuvettes (1 cm light path) containing appropriate solutions in 1 mL PBS, pH 6.8, using Beckman's DU 650 spectrophotometer at room temperature (25 °C).
