# Supplementary material for: Rose Bengal-Mediated Photoinactivation of Multidrug Resistant Pseudomonas aeruginosa Is Enhanced in the Presence of Antimicrobial Peptides
Source: Front Microbiol. 2018 Aug 20;9:1949. doi: 10.3389/fmicb.2018.01949 (PMC6110182; doi:10.3389/fmicb.2018.01949)
Supplement: TABLE S1 — Numerical values of HaCaT cell viability assay (MTT assay) results are presented in Figure 6. [file Table_1.DOCX]

| Phototoxicity | absorbance (aPDI TREATMENT) | | | | | | | | |
| --- | --- | --- | --- | --- | --- | --- | --- | --- | --- |
|  | **0 μM** | **5 μM RB** | **10 μM RB** | **5 μM RB+ 5 μM PEX** | **10 μM RB + 10 μM PEX** | **10 μM PEX** | **5 μM RB + 5 μM CAMEL** | **10 μM RB + 10 μM CAMEL** | **10 μM CAMEL** |
|  | 0.197705 | 0.204416 | 0.200492 | 0.182017419 |  | 0.176632127 | 0.200730558 | 0.180377313 | 0.163055795 |
|  | 0.190594 | 0.207839 | 0.190502 | 0.204921285 | 0.117039286 | 0.199251913 | 0.195875434 | 0.195173461 | 0.191372472 |
|  | 0.193555 | 0.195834 | 0.2050517 | 0.180154121 | 0.101660365 | 0.19572346 | 0.192612082 | 0.183904312 | 0.191220504 |
|  | 0.244943 | 0.194671 | 0.1973302 | 0.191685799 |  | 0.274494746 | 0.197257038 | 0.163141295 | 0.196094012 |
|  | 0.252909 | 0.207477 | 0.1957297 | 0.194492968 | 0.243453651 | 0.275116584 | 0.17060576 | 0.186058319 | 0.184670153 |
|  | 0.250813 | 0.188335 | 0.2025635 |  | 0.239214505 | 0.254155735 | 0.167786093 | 0.177578392 | 0.194538769 |
| mean value | **0.221753** | **0.199762** | **0.1986115** | **0.190654318** | **0.175341952** | **0.229229094** | **0.187477827** | **0.181038849** | **0.186825284** |
| Survival | **100%** | **90.08%** | **89.56%** | **85.98%** | **79.07%** | **103.37%** | **84.54%** | **81.64%** | **84.25%** |
| SD | **0.030651** | **0.007979** | **0.0052219** | **0.01005231** | **0.076478938** | **0.043734833** | **0.014426221** | **0.010638872** | **0.012288492** |
|  |  |  |  |  |  |  |  |  |  |
| Cytotoxicity | absorbance (DARK) | | | | | | | | |
|  | **0 μM** | **5 μM RB** | **10 μM RB** | **5 μM RB+ 5 μM PEX** | **10 μM RB + 10 μM PEX** | **10 μM PEX** | **5 μM RB + 5 μM CAMEL** | **10 μM RB + 10 μM CAMEL** | **10 μM CAMEL** |
|  | 0.175462 | 0.203038 | 0.2094743 | 0.201421529 | 0.185782402 | 0.253798402 | 0.218386392 | 0.186722793 | 0.180025591 |
|  | 0.193724 | 0.21388 | 0.2097983 | 0.21870196 | 0.213944616 | 0.246035909 | 0.195556181 | 0.191699011 | 0.174068083 |
|  | 0.193513 | 0.191305 | 0.201229 | 0.205286178 | 0.192266355 | 0.254960873 | 0.238089912 | 0.174342854 | 0.159958101 |
|  | 0.246489 | 0.185553 | 0.1835683 | 0.241722385 | 0.243960148 | 0.197082413 | 0.210337354 | 0.154001522 | 0.195741163 |
|  | 0.255822 | 0.203227 | 0.2078623 | 0.219579488 | 0.245911478 | 0.187387001 | 0.211312153 | 0.155752116 | 0.193763879 |
|  | 0.26464 | 0.226468 | 0.212939 | 0.214189959 |  | 0.168842306 | 0.203041639 | 0.15166697 | 0.196094012 |
| mean value | **0.221608** | **0.203912** | **0.2041452** | **0.216816917** | **0.216373** | **0.218017817** | **0.212787272** | **0.169030878** | **0.183275138** |
| Survival | **100%** | **92.01%** | **92.12%** | **97.84%** | **97.64%** | **98.38%** | **96.02%** | **76.27%** | **82.70%** |
| SD | **0.038309** | **0.014869** | **0.010802** | **0.014201017** | **0.028090855** | **0.038012687** | **0.014639943** | **0.0176566** | **0.01462021** |
